# Cigarette smoking and risk of gestational diabetes: a systematic review of observational studies

**DOI:** 10.1186/1471-2393-8-53

**Published:** 2008-12-16

**Authors:** Eliana M Wendland, Maria Eugênia Pinto, Bruce B Duncan, José M Belizán, Maria Inês Schmidt

**Affiliations:** 1 Graduate Studies Program in Epidemiology, Faculty of Medicine, Federal University of Rio Grande do Sul, Porto Alegre, Brazil; 2 Department of Mother & Child Health Research, Institute for Clinical Effectiveness and Health Policy (IECS), Buenos Aires, Argentina

## Abstract

**Background:**

Gestational diabetes is a prevalent disease associated with adverse outcomes of pregnancy. Smoking as been associated with glucose intolerance during pregnancy in some but not all studies. Therefore, we aimed to systematically review all epidemiological evidence to examine the association between cigarette smoking during pregnancy and risk of developing gestational diabetes mellitus.

**Methods:**

We conducted a systematic review of articles published up to 2007, using PubMed, Embase, LILACS e CINAHL to identify the articles. Because this review focuses on studies of smoking during pregnancy, we excluded studies evaluating smoking outside pregnancy. Two investigators independently abstracted information on participant's characteristics, assessment of exposure and outcome, and estimates for the association under study. We evaluated the studies for publication bias and performed heterogeneity analyses. We also assessed the effect of each study individually through sensitivity analysis.

**Results:**

We found and critically reviewed 32 studies, of which 12 met the criteria for inclusion in the review. Most of the studies provided only unadjusted measurements. Combining the results of the individual studies, we obtained a crude odds ratio of 1.03 (99% CI 0.85–1.25). Only 4 studies presented adjusted measurements of association, and no association was found when these alone were analyzed (OR 0.95; 99% CI 0.85–1.07). Subgroup analysis could not be done due to small sample size.

**Conclusion:**

The number of studies is small, with major heterogeneity in research design and findings. Taken together, current data do not support an association between cigarette smoking during pregnancy and the risk of gestational diabetes.

## Background

Gestational diabetes mellitus (GDM) is defined as glucose intolerance with onset or first recognition during pregnancy [1], and affects 1 to 15% of all pregnancies overall [2] and 7.6% of pregnancies in Brazil [3]. The prevalence of gestational diabetes mellitus varies in direct proportion with the prevalence of type 2 diabetes and is increasing over time along with the prevalence of obesity [4]. Its onset is associated with increased rates of macrosomia which in turn increases the risk of cesarean section, shoulder dystocia and birth trauma [3,5,6]. A long-term consequence of gestational diabetes for the mother is increased risk of progression to type 2 diabetes later in life [7,8].

Many predisposing factors, such as advanced age, obesity, non-Caucasian ethnicity, and family history of type 2 diabetes have been associated with an increased risk of gestational diabetes [9,10]. Previous gestational diabetes, unexplained fetal loss or newborns large for gestational age have also been linked to an increased risk of gestational diabetes mellitus [11].

Although a tendency of reduction in the number of women who smoke during pregnancy has been observed [12], cigarette smoking is still common during pregnancy [13-16]. Smoking during pregnancy has been associated with short and long term adverse outcomes including premature rupture of fetal membranes, placenta previa, placental abruption [17], preterm delivery [18], and future childhood obesity and hypertension [19]. Increased insulin resistance [20,21], hyperinsulinemia and type 2 diabetes have been linked with cigarette smoking outside of pregnancy in some but not all studies [18,22], but whether cigarette smoking is a risk factor for the development of gestational diabetes remains controversial [23,24]. Intriguingly, smoking has been negatively associated with pre-eclampsia [25] and, more recently, outside of pregnancy, with reduced risk of some gastrointestinal diseases [26] and Parkinson's disease [27].

Given this uncertainty about the relationship between smoking in pregnancy and GDM, the aim of this study is to systematically review all epidemiological evidence on the relation between smoking habits during pregnancy and risk of gestational diabetes mellitus.

## Methods

A specific protocol was designed for this systematic review, which was reported in accordance with the checklist proposed by the Meta-analysis of Observational Studies in Epidemiology (MOOSE) group [28].

### Search strategies

We searched for published and unpublished studies reported from 1970 to 2006 in PubMed . The electronic search strategy was constructed using the key terms suggested by the Metabolic and Endocrine Disorders Group [29], Pregnancy and Childbirth Group [30] and Tobacco Addiction Group [31]: "smoke" or "smoking" or "tobacco use" or "cigarette" combined with "gestational diabetes" or ("diabetes" and "pregnancy") in text words or medical subject headings. We used similar strategies to search LILACS (Latin America and Caribbean database), EMBASE and CINAHL. Additionally, to avoid publication bias, we did a broader search, generically using the term "risk factor", looking for articles in which smoking was not necessarily the primary exposure. We manually searched reference lists of retrieved articles and of relevant reviews, as well as web pages of selected ministries of health and other potentially relevant internet sources. No attempt was made to contact the authors of any of these studies in order to get primary data. We restricted our search to studies of humans, with no language restrictions.

### Criteria for considering studies

We considered all observational studies that assessed the association between smoking cigarettes during pregnancy and gestational diabetes, and which provided adjusted or crude relative risks (RR), odds ratios (OR) or information that enabled us to calculate the crude measures of association. The diagnosis of gestational diabetes had to be obtained by an oral glucose tolerance test (OGTT) or by a clinical diagnosis. We excluded studies of type 1 diabetes, reports of tobacco products other than cigarettes, animal studies and case series. Studies not having a clear statement that smoking occurred during pregnancy, not having exposure measured before the outcome, or not having a clear definition of the diagnostic process for the outcome were not included.

### Study selection and data extraction

Titles and abstracts of the resulting publications were screened for articles of possible interest by two independent investigators (E.M.W. – obstetrician and M.E.P. – primary care physician). When the information provided by the title and abstract was not sufficient to determine exclusion, we evaluated the full-text. For data extraction, we adapted a form recommended by Cochrane Non-Randomized Studies Methods Group [32]. Two investigators independently abstracted information on participant characteristics, measurements of smoking habits and outcomes, adjustment for potential confounders, and estimates of association. Discrepancies were resolved by discussion and repeated examination of the articles and, when necessary, through consultation of a third author (M.I.S.).

### Appraisal of methodological quality of primary studies

All articles meeting the eligibility criteria were assessed for their methodological quality by two independent investigators (E.M.W. and M.E.P). This assessment involved scrutinizing study design, sampling method, source of data and definition of exposure and diagnostic procedures. The presence of clear definitions of exposure and diagnostic methods were regarded as an indication of higher quality.

### Data synthesis and statistical analysis

We used MIX – Meta-analysis with Interactive Explanations (version 1.54) [33] for all statistical analyses. When an unadjusted odds ratio (OR) for smoking – gestational diabetes mellitus association was not provided in the manuscript, we manually calculated it from the data provided. Data input was double-checked for accuracy, and the ORs calculated by MIX were compared to the ORs reported in the original studies.

Combination of results involved inverse-variance-weighted averages of the log odds ratio [ln(OR)]. Initially, the overall association was calculated using a Mantel-Haenzel fixed-effects model. Heterogeneity was assessed using Cochran's Q test. As this test is considered to have low statistical power, especially when the number of studies included in the meta-analysis is small (< 20), a random effect model (DerSimonian-Laird) was used if the *P*-value was less than 0.1 [34]. Confidence intervals of 99% were used for individual and overall associations to allow for the increased possibility of random error in multiple comparisons. Meta-analysis was applied to crude odds ratios, and, when available, additionally to adjusted odds ratios. Degree of adjustment for potential confounders were categorized as "+" for age; "++" for age plus BMI; "+++" for these plus weight gain during pregnancy.

Publication bias was evaluated quantitatively using Egger's regression, in which the standardized effect estimates are regressed against estimated precision [35]. We did not employ graphical methods such as the funnel plot as this technique requires a larger number of studies to provide an adequate graph [36]. To investigate the impact of the control for confounding factors on the study estimates, we grouped the studies according with the presence or absence of adjustments.

In addition, to evaluate the stability of the results of this meta-analysis and to explore the effect of heterogeneity in studies, we also performed a one-way sensitivity analysis. By removing one study at a time and recalculating the odds ratio, we observed the effect of each study on the summary estimate, thus evaluating the robustness of the results [35]. When possible, additional meta-analyses were performed on relevant sub-groups of studies to investigate possible causes of heterogeneity.

## Results

**Figure 1:**
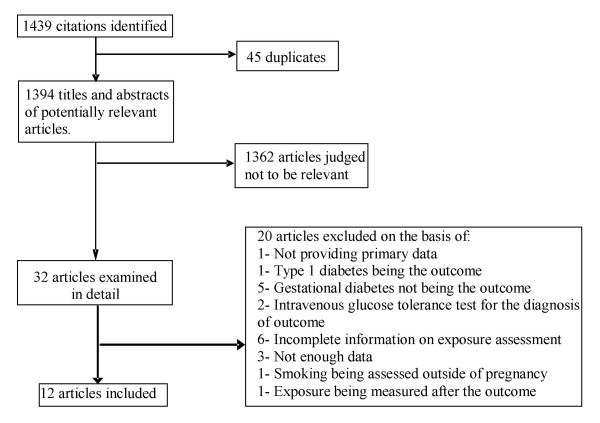
**Flow diagram of the study selection process summarizing study assessment and exclusion stages**.

We initially identified 1439 references, 1354 in Medline, 68 in EMBASE, 9 in LILACS and 8 in CINAHL. After exclusions determined by abstract review, 32 studies were considered. Of these, we excluded an additional 20 studies, 12 thus remaining for the systematic review (Figure 1). These exclusions were due to not assessing smoking during pregnancy [37]; not having a clear definition of the diagnostic process [38-43] or diagnosing GDM with intravenous tolerance testing [44,45]; lacking sufficient information to calculate measures of association [46-48]; not providing primary data [49]; not ascertaining GDM [50-55]; or not assessing the exposure before the outcome [56].

Table 1 shows selected characteristics of the studies analyzed. Two studies that investigated the same study population [24,57], but with somewhat different sample size and results, were included. All studies that provided adjusted measurements included age in the analysis.

**Table 1:** One-way sensitivity analysis.

Study excluded	Random effects model	
	Odds ratio	99% CI
Wendland et al[59]	1.03	0.72–1.48
England et al. [24]	0.94	0.84–1.06
Xiong et al. [60]	0.95	0.85–1.07
Rodrigues et al. Cree [66]	0.96	0.85–1.07
Rodrigues et al. Non-native [66]	0.95	0.85–1.07

As shown in Additonal File 1, universal and selective screening (or both) for gestational diabetes was used. Four studies used the 2-h OGTT (WHO definition), seven used the 3-h OGTT (ADA definition), one used more than one criteria for diagnosis of GDM and another used the ICD-code for gestational diabetes. Studies varied in their classification of smoking status. For example, some separated women who quit smoking before and during pregnancy. Others pooled all women who smoked any cigarette during pregnancy. Only three studies provided measurements of association for different smoking categories [23,24,58]. All studies were based on self-report of smoking. None of the studies evaluated in this systematic review provided information about the type of cigarettes smoked.

**Figure 2:**
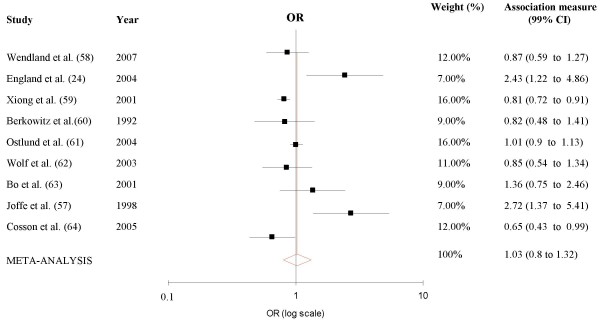
**Meta-analysis of unadjusted results of studies of the association between smoking and gestational diabetes**. Black squares indicate the odds ratio in each study and the horizontal lines represent 99% confidence intervals. Random-effects model.

As significant heterogeneity between studies was found in the meta-analysis of the nine [24,57,59-65] reports of crude associations (Q = 50.14; p < 0.01), we used a random effects method for this analysis (Figure 2). No association between smoking during pregnancy and GDM was present in such analysis, the summary unadjusted OR being 1.03 (99% CI 0.85–1.25).

**Figure 3:**
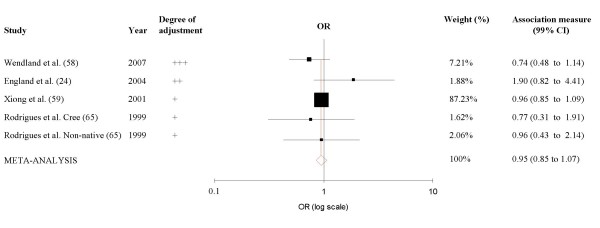
**Meta-analysis of adjusted results of studies of the association between smoking during pregnancy and gestational diabetes**. Black squares indicate the odds ratio in each study, with the square size proportional to the weight of the study in the meta-analysis and the horizontal lines represent 99% confidence intervals. Fixed-effects model.

Only four studies, including five distinct populations, described adjusted odds ratios and assessed potential confounders when non smokers were compared to current smokers [24,59,66,67]. As heterogeneity between studies did not reach statistical significance in the adjusted meta-analysis (Q = 7.0; p = 0.14), we used a fixed effects model. Combining the adjusted results produced an overall odds ratio of 0.95 (99% CI 0.85–1.07; p = 0.27) (Figure 3). Since the native Cree Canadian population has a high prevalence of gestational diabetes [68], we conducted a sensitivity analysis excluding this population; only minimal changes were found in the summary measurement (OR = 0.97; 95% CI 0.69–1.38). Sub-group analysis by type of diagnostic criteria (3-hour OGTT versus 2-h OGTT), did not reveal important differences. Additional sub-group analysis by gestational age of smoking assessment showed that when smoking was assessed at less than 24 weeks of pregnancy [24,57,62,63,65], the combined crude OR, using a random effects model, was 1.22 (99% CI 0.73–2.04), whereas when smoking was assessed at later than 24 weeks [59,61,64,69], it was 0.88 (99% CI 0.70–1.10).

We identified three studies, assessing different levels of smoking [23,24,70] (Additonal File 1). A fixed effects analysis showed no association of light smoking (1–9 cig/day) with GDM (OR = 0.94; 99% CI 0.83–1.06), without heterogeneity between studies (Q = 4.25; p = 0.24). Only two studies [23,58] presented comparable categories for heavy smokers (more than 10 cig/day). In a random effects model, given that significant heterogeneity was present (Q = 5.66; p = 0.06), similar results were found for women that smoked 10 or more than cigarettes per day during pregnancy (OR = 0.72; 99% CI 0.37–1.42).

The Egger's test provided no evidence of publication bias for the unadjusted (p = 0.31) or adjusted (p = 0.92) overall associations of smoking with gestational diabetes. In the sensitivity analysis, the overall heterogeneity and association size were recalculated by iteratively removing one study at a time. This analysis confirmed the stability of the summary risk estimate (Additonal File 2).

## Discussion

Cigarette smoking has been considered as a risk factor for diabetes outside of pregnancy [20,71] and as such, could also be seen as a risk factor for gestational diabetes [24,37]. However, our meta-analysis does not support the hypothesis that smoking during pregnancy increases the risk of gestational diabetes.

To our knowledge, this is the first systematic review and meta-analysis evaluating the association of smoking during pregnancy and gestational diabetes. Although we found a great diversity in the assessment of outcomes and adjustment for confounding variables, sensitivity analysis did not reveal an important influence of any single study (Additonal File 2). As our results are based on published studies only, other small studies describing a null association may have escaped identification.

The evidence published so far for the association between smoking and gestational diabetes is inconsistent [23,24,60,66]. How to reconcile these differences is not clear. It is possible that differences between the study settings such as screening procedures for GDM, or due to variations in the content of cigarettes [72] or in the frequency of stopping smoking during pregnancy may explain this inconsistency. Lumping ex-smokers with never smokers could raise the risk in this comparison group, producing an apparent lower risk in smokers. However the only two studies explicitly reporting data on quitters in the adjusted models showed inconsistent results [59,73].

One such difference meriting special consideration is the moment of measuring smoking during pregnancy. Smoking cessation or reduction in the number of cigarettes smoked during pregnancy accentuates gestational weight gain [74], an important risk factor for gestational diabetes [75]. Thus, failure to identify smokers who quit after ascertainment or who reduced the number of cigarettes smoked during pregnancy may lead to information bias, erroneously attributing the association found to smoking rather than to its reduction. Furthermore, social pressure to quit smoking may lead to erratic smoking behavior during pregnancy, difficult to access in epidemiologic studies [76]. In this regard, our meta-analysis of reports that assessed smoking earlier in pregnancy [24,57,62,63,65] showed a tendency to present an increased risk of developing gestational diabetes, while our meta-analysis of studies that ascertained smoking later in pregnancy, and thus possibility more accurately, [59-61,66] showed odds ratios slightly less than 1.

Our study illustrates the difficulties of systematic reviews of observational studies. We identified a variety of definitions in the ascertainment of gestational diabetes and part of the variation in the results between studies may be related to this variation. Moreover, the degree of information in the reports is frequently less than desirable. Aspects such as characteristics of the population, definitions of exposures and of diagnostic procedures and thus outcomes, statistical analysis routines and measures of association are not systematically described in the reports, limiting the comparability of the studies and utility of some of the extracted data. The inclusion of two studies [24,57] referring to the same population in the crude analysis may have biased the crude estimate slightly upward. However, this does not appear to have been an important problem as the adjusted analysis, in which only one of these studies was included, showed a similar result.

Other limitations of the present meta-analysis must be considered. The small number of reports published did not allow us to do extensive sensitivity nor subgroup analyses. Another potential limitation, as previously mentioned, is information bias with respect to categorization of cigarette smoking, as all studies were based on a limited assessment by self-reports of smoking habits. As women in general tend to under-report smoking during pregnancy by about 15% [77], non-differential misclassification could bias the results to the null.

## Conclusion

Current data demonstrate important heterogeneity and, when taken together, do not support an association between cigarette smoking during pregnancy and the risk of gestational diabetes. Further research with more detailed and objective measurements of smoking is needed to evaluate this association. In vitro and animal studies may further help to clarify possible biologic mechanisms and pathways by which cigarette smoking may play a role in the development of gestational diabetes.

## Competing interests

The authors declare that they have no competing interests.

## Authors' contributions

EMW carried out the search, acquisition and interpretation of the data in studies, performed the statistical analysis and drafted the manuscript. MEP participated in the selection of studies; MIS participated in design of the study, selection of studies and manuscript preparation; BBD participated in the design of the study and manuscript preparation; JMB participated in the final version of the study. All authors read and approved the final manuscript.

## Pre-publication history

The pre-publication history for this paper can be accessed here:



## Supplementary Material

Additional file 1 **Characteristics of studies of the association of cigarette smoking and gestational diabetes mellitus.** Click here for file

Additional file 2 **Summary of study diagnostic process characteristics and associations found between smoking and gestational diabetes.** Click here for file

## References

[B1] Metzger BE, Coustan DR (1998). Summary and recommendations of the Fourth International Workshop-Conference on Gestational Diabetes Mellitus. The Organizing Committee. Diabetes Care.

[B2] King H (1998). Epidemiology of glucose intolerance and gestational diabetes in women of childbearing age. Diabetes Care.

[B3] Schmidt MI, Duncan BB, Reichelt AJ, Branchtein L, Matos MC, Costa e Forti (2001). Gestational diabetes mellitus diagnosed with a 2-h 75-g oral glucose tolerance test and adverse pregnancy outcomes. Diabetes Care.

[B4] Dabelea D, Snell-Bergeon JK, Hartsfield CL, Bischoff KJ, Hamman RF, McDuffie RS (2005). Increasing prevalence of gestational diabetes mellitus (GDM) over time and by birth cohort: Kaiser Permanente of Colorado GDM Screening Program. Diabetes Care.

[B5] Jarrett RJ, Castro-Soares J, Dornhorst A, Beard RW, Castro-Soares J (1997). Should we screen for gestational diabetes?. BMJ.

[B6] Sermer M, Naylor CD, Farine D, Kenshole AB, Ritchie JW, Gare DJ (1998). The Toronto Tri-Hospital Gestational Diabetes Project. A preliminary review. Diabetes Care.

[B7] Jacob Reichelt AA, Ferraz TM, Rocha Oppermann ML, Costa e Forti, Duncan BB, Fleck PE (2002). Detecting glucose intolerance after gestational diabetes: inadequacy of fasting glucose alone and risk associated with gestational diabetes and second trimester waist-hip ratio. Diabetologia.

[B8] Cheung NW, Byth K (2003). Population health significance of gestational diabetes. Diabetes Care.

[B9] Naylor CD, Sermer M, Chen E, Farine D (1997). Selective screening for gestational diabetes mellitus. Toronto Trihospital Gestational Diabetes Project Investigators. N Engl J Med.

[B10] Davey RX, Hamblin PS (2001). Selective versus universal screening for gestational diabetes mellitus: an evaluation of predictive risk factors. Med J Aust.

[B11] Alberti KG, Zimmet PZ (1998). Definition, diagnosis and classification of diabetes mellitus and its complications. Part 1: diagnosis and classification of diabetes mellitus provisional report of a WHO consultation. Diabet Med.

[B12] CDC (2004). Smoking during pregnancy – United States, 1990–2002. MMWR Morb Mortal Wkly Rep.

[B13] (1998). ACOG educational bulletin. Smoking and women's health. Number 240, September 1997 (Replaces No. 180, May 1993). American College of Obstetricians and Gynecologists. Int J Gynaecol Obstet.

[B14] Kaneita Y, Tomofumi S, Takemura S, Suzuki K, Yokoyama E, Miyake T (2007). Prevalence of smoking and associated factors among pregnant women in Japan. Prev Med.

[B15] Ward C, Lewis S, Coleman T (2007). Prevalence of maternal smoking and environmental tobacco smoke exposure during pregnancy and impact on birth weight: retrospective study using Millennium Cohort. BMC Public Health.

[B16] Kroeff LR, Mengue SS, Schmidt MI, Duncan BB, Favaretto AL, Nucci LB (2004). [Correlates of smoking in pregnant women in six Brazilian cities]. Rev Saude Publica.

[B17] Castles A, Adams EK, Melvin CL, Kelsch C, Boulton ML (1999). Effects of smoking during pregnancy. Five meta-analyses. Am J Prev Med.

[B18] U.S. Department of Health and Human Services (2001). Women and smoking: A report of the Surgeon General.

[B19] Oken E, Huh SY, Taveras EM, Rich-Edwards JW, Gillman MW (2005). Associations of maternal prenatal smoking with child adiposity and blood pressure. Obes Res.

[B20] Rimm EB, Manson JE, Stampfer MJ, Colditz GA, Willett WC, Rosner B (1993). Cigarette smoking and the risk of diabetes in women. Am J Public Health.

[B21] Will JC, Galuska DA, Ford ES, Mokdad A, Calle EE (2001). Cigarette smoking and diabetes mellitus: evidence of a positive association from a large prospective cohort study. Int J Epidemiol.

[B22] Meigs JB, Nathan DM, Cupples LA, Wilson PW, Singer DE (1996). Tracking of glycated hemoglobin in the original cohort of the Framingham Heart Study. J Clin Epidemiol.

[B23] Terry PD, Weiderpass E, Ostenson CG, Cnattingius S (2003). Cigarette smoking and the risk of gestational and pregestational diabetes in two consecutive pregnancies. Diabetes Care.

[B24] England LJ, Levine RJ, Qian C, Soule LM, Schisterman EF, Yu KF (2004). Glucose tolerance and risk of gestational diabetes mellitus in nulliparous women who smoke during pregnancy. Am J Epidemiol.

[B25] Conde-Agudelo A, Althabe F, Belizan JM, Kafury-Goeta AC (1999). Cigarette smoking during pregnancy and risk of preeclampsia: a systematic review. Am J Obstet Gynecol.

[B26] Thomas GA, Rhodes J, Ingram JR (2005). Mechanisms of disease: nicotine – a review of its actions in the context of gastrointestinal disease. Nat Clin Pract Gastroenterol Hepatol.

[B27] Thacker EL, O'Reilly EJ, Weisskopf MG, Chen H, Schwarzschild MA, McCullough ML (2007). Temporal relationship between cigarette smoking and risk of Parkinson disease. Neurology.

[B28] Stroup DF, Berlin JA, Morton SC, Olkin I, Williamson GD, Rennie D (2000). Meta-analysis of observational studies in epidemiology: a proposal for reporting. Meta-analysis Of Observational Studies in Epidemiology (MOOSE) group. JAMA.

[B29] Richter B, Bergerhoff K, Paletta G, Bandeira-Echtler E (2007). Metabolic and Endocrine Disorders Group.

[B30] The Editorial Team (2007). Pregnancy and Childbirth Group. Issue 4 edn.

[B31] Lancaster T, Stead T, Cahill K, West R, Aveyard P, Hughes J (2007). Tobacco Addiction Group. Issue 4 edn.

[B32] Olsen O, Shea B, Wells G (2005). Extracting data.

[B33] Bax L, Yu LM, Ikeda N, Tsuruta H, Moons KG (2006). Development and validation of MIX: comprehensive free software for meta-analysis of causal research data. BMC Med Res Methodol.

[B34] DerSimonian R, Laird N (1986). Meta-analysis in clinical trials. Control Clin Trials.

[B35] Egger M, Davey SG, Schneider M, Minder C (1997). Bias in meta-analysis detected by a simple, graphical test. BMJ.

[B36] NHMRC (1999). How to review the evidence: systematic identification and review of the scientific literature.

[B37] Solomon CG, Willett WC, Carey VJ, Rich-Edwards J, Hunter DJ, Colditz GA (1997). A prospective study of pregravid determinants of gestational diabetes mellitus. JAMA.

[B38] Retnakaran R, Hanley AJ, Raif N, Connelly PW, Sermer M, Zinman B (2003). C-reactive protein and gestational diabetes: the central role of maternal obesity. J Clin Endocrinol Metab.

[B39] Heckbert SR, Stephens CR, Daling JR (1988). Diabetes in pregnancy: maternal and infant outcome. Paediatr Perinat Epidemiol.

[B40] Rudra CB, Williams MA, Lee IM, Miller RS, Sorensen TK (2006). Perceived exertion in physical activity and risk of gestational diabetes mellitus. Epidemiology.

[B41] Yang X, Hsu-Hage B, Zhang H, Yu L, Dong L, Li J (2002). Gestational diabetes mellitus in women of single gravidity in Tianjin City, China. Diabetes Care.

[B42] Kieffer EC, Sinco B, Kim C (2006). Health behaviors among women of reproductive age with and without a history of gestational diabetes mellitus. Diabetes Care.

[B43] van Hoorn J, Dekker G, Jeffries B (2002). Gestational diabetes versus obesity as risk factors for pregnancy-induced hypertensive disorders and fetal macrosomia. Aust N Z J Obstet Gynaecol.

[B44] Goldman JA, Schechter A (1967). Effect of cigarette smoking on glucose tolerance in pregnant women. Isr J Med Sci.

[B45] Langhoff-Roos J, Wibell L, Gebre-Medhin M, Lindmark G (1993). Effect of smoking on maternal glucose metabolism. Gynecol Obstet Invest.

[B46] Zaren B, Lindmark G, Wibell L, Folling I (2000). The effect of smoking on glucose homeostasis and fetal growth in pregnant women. Ups J Med Sci.

[B47] Saldana TM, Siega-Riz AM, Adair LS (2004). Effect of macronutrient intake on the development of glucose intolerance during pregnancy. Am J Clin Nutr.

[B48] Gohdes D, Oser CS, Harwell TS, Moore KR, McDowall JM, Helgerson SD (2004). Diabetes in Montana's Indians: the epidemiology of diabetes in the Indians of the Northern Plains and Canada. Curr Diab Rep.

[B49] Dornhorst A, Rossi M (1998). Risk and prevention of type 2 diabetes in women with gestational diabetes. Diabetes Care.

[B50] Nylund L, Lunell NO, Persson B, Fredholm BB, Lagercrantz H (1988). Smoking exerts a ketogenic influence in diabetic pregnancy. Gynecol Obstet Invest.

[B51] Ros HS, Cnattingius S, Lipworth L (1998). Comparison of risk factors for preeclampsia and gestational hypertension in a population-based cohort study. Am J Epidemiol.

[B52] Montgomery SM, Ekbom A (2002). Smoking during pregnancy and diabetes mellitus in a British longitudinal birth cohort. BMJ.

[B53] Caulfield LE, Harris SB, Whalen EA, Sugamori ME (1998). Maternal nutritional status, diabetes and risk of macrosomia among Native Canadian women. Early Hum Dev.

[B54] Rosenberg MS (2005). The file-drawer problem revisited: a general weighted method for calculating fail-safe numbers in meta-analysis. Evolution.

[B55] Bottini E, Gloria-Bottini F, La Torre M, Lucarini N (2001). The genetics of signal transduction and the effect of smoking on intrauterine growth. Int J Epidemiol.

[B56] Dempsey JC, Butler CL, Sorensen TK, Lee IM, Thompson ML, Miller RS (2004). A case-control study of maternal recreational physical activity and risk of gestational diabetes mellitus. Diabetes Res Clin Pract.

[B57] Joffe GM, Esterlitz JR, Levine RJ, Clemens JD, Ewell MG, Sibai BM (1998). The relationship between abnormal glucose tolerance and hypertensive disorders of pregnancy in healthy nulliparous women. Calcium for Preeclampsia Prevention (CPEP) Study Group. Am J Obstet Gynecol.

[B58] Cnattingius S, Lambe M (2002). Trends in smoking and overweight during pregnancy: prevalence, risks of pregnancy complications, and adverse pregnancy outcomes. Semin Perinatol.

[B59] Wendland EM (2007). O hábito de fumar e o risco de desenvolver diabetes e hipertensão durante a gestação. Graduate Studies Program in Epidemiology.

[B60] Xiong X, Saunders LD, Wang FL, Demianczuk NN (2001). Gestational diabetes mellitus: prevalence, risk factors, maternal and infant outcomes. Int J Gynaecol Obstet.

[B61] Berkowitz GS, Lapinski RH, Wein R, Lee D (1992). Race/ethnicity and other risk factors for gestational diabetes. Am J Epidemiol.

[B62] Ostlund I, Haglund B, Hanson U (2004). Gestational diabetes and preeclampsia. Eur J Obstet Gynecol Reprod Biol.

[B63] Wolf M, Sandler L, Hsu K, Vossen-Smirnakis K, Ecker JL, Thadhani R (2003). First-trimester C-reactive protein and subsequent gestational diabetes. Diabetes Care.

[B64] Bo S, Menato G, Lezo A, Signorile A, Bardelli C, De Michieli F (2001). Dietary fat and gestational hyperglycaemia. Diabetologia.

[B65] Cosson E, Benchimol M, Carbillon L, Pharisien I, Paries J, Valensi P (2006). Universal rather than selective screening for gestational diabetes mellitus may improve fetal outcomes. Diabetes Metab.

[B66] Rodrigues S, Robinson EJ, Ghezzo H, Gray-Donald K (1999). Interaction of body weight and ethnicity on risk of gestational diabetes mellitus. Am J Clin Nutr.

[B67] Xiong X, Wang FL, Davidge ST, Demianczuk NN, Mayes DC, Olson DM (2000). Maternal smoking and preeclampsia. J Reprod Med.

[B68] Rodrigues S, Robinson E, Gray-Donald K (1999). Prevalence of gestational diabetes mellitus among James Bay Cree women in northern Quebec. CMAJ.

[B69] Wendland EM, Duncan BB, Mengue SS, Nucci LB, Schmidt MI (2007). Waist circumference in the prediction of obesity-related adverse pregnancy outcomes. Cad Saude Publica.

[B70] Cnattingius S (2004). The epidemiology of smoking during pregnancy: smoking prevalence, maternal characteristics, and pregnancy outcomes. Nicotine Tob Res.

[B71] Wannamethee SG, Shaper AG, Perry IJ (2001). Smoking as a modifiable risk factor for type 2 diabetes in middle-aged men. Diabetes Care.

[B72] Dooley MA, Hogan SL (2003). Environmental epidemiology and risk factors for autoimmune disease. Curr Opin Rheumatol.

[B73] England LJ, Levine RJ, Qian C, Morris CD, Sibai BM, Catalano PM (2002). Smoking before pregnancy and risk of gestational hypertension and preeclampsia. Am J Obstet Gynecol.

[B74] Favaretto AL, Duncan BB, Mengue SS, Nucci LB, Barros EF, Kroeff LR (2007). Prenatal weight gain following smoking cessation. Eur J Obstet Gynecol Reprod Biol.

[B75] Kabiru W, Raynor BD (2004). Obstetric outcomes associated with increase in BMI category during pregnancy. Am J Obstet Gynecol.

[B76] Pickett KE, Wakschlag LS, Dai L, Leventhal BL (2003). Fluctuations of maternal smoking during pregnancy. Obstet Gynecol.

[B77] Klebanoff MA, Levine RJ, Morris CD, Hauth JC, Sibai BM, Ben Curet L (2001). Accuracy of self-reported cigarette smoking among pregnant women in the 1990s. Paediatr Perinat Epidemiol.

