# Supplementary material for: Cigarette smoking and risk of gestational diabetes: a systematic review of observational studies
Source: BMC Pregnancy Childbirth. 2008 Dec 16;8:53. doi: 10.1186/1471-2393-8-53 (PMC2632653; doi:10.1186/1471-2393-8-53)
Supplement: Additional file 1 — Characteristics of studies of the association of cigarette smoking and gestational diabetes mellitus. [file 1471-2393-8-53-S1.doc]

Additional file 1 - **Characteristics of studies of the association of cigarette smoking and gestational diabetes mellitus.**

| Author, year | Location | Data source | Description of the study population | Adjustments | Degree of adjustment* |
| --- | --- | --- | --- | --- | --- |
| Terry et al., 2003 [23] | Sweden | Birth registry | First and second deliveries between January, 1987 and December, 1995. | Maternal age, living with infant’s father, interpregnancy interval, maternal height, BMI during pregnancy. | ++ |
|  |  |  |  |  |  |
| England et al., 2004 [24] | U.S. | CPEP trial | Participants of the Calcium for Pre-eclampsia Prevention trial. 1992 to 1995. | Race/ethnicity, age, education, BMI at enrollment, pregnancy loss, private health insurance, study center, gestational age at blood collection. | ++ |
|  |  |  |  |  |  |
| Cnattingius et al., 2002 [69] | Sweden | Birth register | Women with singleton births between 1992 and 1997, in Sweden. | Age, parity, cohabitation with infant’s father, maternal education, mother’s country of birth, maternal height, BMI at first antenatal care visit. | ++ |
|  |  |  |  |  |  |
| Xiong et al., 2001[60] | Canada | Hospital register | Women from the Northern and Central Alberta Perinatal Audit and Education Program of Canada from July, 1991 to December, 1997. | Parity, age, maternal weight, alcohol use, history of neonatal death, history of delivery < 37 weeks, history of cesarean section and history of major neonatal malformation. | + |
|  |  |  |  |  |  |
| Wendland et al., 2007 [59] | Brazil | Prospective study | Women using the public health clinics in six state capitals of Brazil from 1991 to 1995. | Study center, age, skin color, pre-pregnancy BMI and weight gain during pregnancy. | +++ |
|  |  |  |  |  |  |
| Rodrigues et al., 1999 [66] | Canada | Medical records | Cree population from James Bay and women delivered at Royal Victoria Hospital in Montreal from 1995 to 1996 and women from the MOND database from 1990 to 1996. | Age, parity, prepregnancy body weight. | + |
|  |  |  |  |  |  |
| Berkowitz et al., 1992 [61] | U.S. | Hospital registry | Singleton pregnancies at Mount Sinai Medical Center (NY) from January, 1987 to December, 1989 | None | - |
|  |  |  |  |  |  |
| Bo et al., 2001 [64] | Italy | Prospective study | Pregnant women attending the Gynecological and Obstetrical Department of the University of Turin from April, 1999 to November, 2000. | None | - |
|  |  |  |  |  |  |
| Cosson et al., 2006 [65] | France | Prospective study | Women who delivered at Jean Verdier Hospital - Bondy, from October, 2000 to December, 2002. | None | - |
|  |  |  |  |  |  |
| Wolf et al., 2003 [63] | U.S. | Prospective study | Participants of the Massachusetts General Hospital Obstetric Maternal Study (MOMS) from September, 1998 to July, 2001. | None | - |
|  |  |  |  |  |  |
| Ostlund et al., 2004 [62] | Sweden | Birth registry | All women with singleton births from 1992 to 1996, registered in the Swedish Medical Birth register. | None | - |
|  |  |  |  |  |  |
| Joffe et al., 1998 [57] | U.S. | CPEP trial | Participants of the Calcium for Preeclampsia Prevention Trial with complete glucose screening. 1992 to 1995. | None | - |

*Degree of adjustment for potential confounders were categorized as + for age; ++ for age plus prepregnancy BMI; +++ for these plus weight gain during pregnancy.
