# Supplementary material for: Cigarette smoking and risk of gestational diabetes: a systematic review of observational studies
Source: BMC Pregnancy Childbirth. 2008 Dec 16;8:53. doi: 10.1186/1471-2393-8-53 (PMC2632653; doi:10.1186/1471-2393-8-53)
Supplement: Additional file 2 — Summary of study diagnostic process characteristics and associations found between smoking and gestational diabetes. [file 1471-2393-8-53-S2.doc]

**Additional file 2 - Summary of study diagnostic process characteristics and associations found between smoking and gestational diabetes.**

| Author, year | Screening characteristics | Diabetes assessment | GDM criteria | Categories of smoking | Crude odds ratio with 95% CI | Adjusted odds ratio with 95% CI | Comments |
| --- | --- | --- | --- | --- | --- | --- | --- |
| Terry et al., 2003 [23] | Selective;  Random blood glucose (≥126mg/dl) in high risk patients (diabetes heredity, previous GDM, overweight, or previous baby >4.5kg) | 2-h 75-g OGTT | WHO  (Fasting ≥ 140mg/dl or 2h ≥ 160mg/dl) and during 1987-1991, a 2h > 140mg/dl). | Non smokers  Smokers  1-9 cig/day  ≥ 10 cig/day | 1.0  0.93 (0.78-1.10) | 1.0  1.10 (0.81-1.49)  1.08 (0.71-1.63) | If smokers are less obese, they are less likely to be screened. As such, investigated smokers may have higher rates of obesity than in universal screening samples. Does not take weight change during pregnancy in account. |
|  |  |  |  |  |  |  |  |
| England et al., 2004 [24] | Universal; 50 g GC according with procedures of individual medical centers (independent of fasting state). Used thresholds: 140mg/dl and 135mg/dl, depending on the medical center. | 3-h 100-g OGTT | National Diabetes Data Group  (F ≥ 105mg/dl, 1h ≥ 190mg/dl, 2h ≥ 165mg/dl or 3h ≥ 145mg/dl)  or  1h 50g ≥ 200mg/dl  Carpenter and Coustan  (>95, 180, 155, 140mg/dL for fasting, 1-hour, 2-hour and 3-hour post OGTT). | Never smoked  Quit before pregnancy  Quit during pregnancy  Smoked  1-9 cig/day  10-19 cig/day  ≥ 20 cig/day  Never smoked  Smoked | 1.0  2.6 (1.5-4.4) | 1.0  0.8 (0.3-2.1)  1.4 (0.6-2.9)  1.9 (1.0-3.6)  1.8 (0.8-3.9)  2.9 (1.2-6.9)  2.1 (0.4-10.2)  1.0  1.4 (0.8-2.5) | Lack of standardization of screening procedure. Only nulliparas were included do not allowing investigation of the effect of parity. Does not take weight change during pregnancy in to account. |
|  |  |  |  |  |  |  |  |
| Cnattingius et al., 2002 [69] | Not stated | Medical records | ICD code – GDM diagnosis using  WHO definition (Fasting ≥ 140mg/dl or 2h ≥ 160mg/dl) | Non-daily smoking  1-9 cig/day  ≥ 10 cig/day |  | 1.0  0.9 (0.8-1.0)  0.9 (0.9-1.1) | Did not report screening procedure. Does not take weight change during pregnancy in to account. |
|  |  |  |  |  |  |  |  |
| Xiong et al., 2001 [60] | Universal;  50-g GC (≥140mg/dl) | 3-h 100-g OGTT | National Diabetes Data Group  (F ≥ 105mg/dl, 1h ≥ 190mg/dl, 2h ≥ 165mg/dl or 3h ≥ 145mg/dl) | Not smoking  Smoking in pregnancy | 1.0  0.81 (0.77-0.92) | 1.0  0.96 (0.87-1.05) | Does not take BMI or weight change during pregnancy in to account. |
|  |  |  |  |  |  |  |  |
| Rodrigues et al., 1999 [66] | Universal; 50-g GC (≥140mg/dl) | 3-h 100-g OGTT | National Diabetes Data Group  (F ≥ 105mg/dl, 1h ≥ 190mg/dl, 2h ≥ 165mg/dl or 3h ≥ 145mg/dl) | Not smoking  Smoking in pregnancy |  | 1.0  0.77 (0.38-1.51) – Cree  0.96 (0.51-1.73) – Non- native | Does not take BMI or weight change during pregnancy in to account. |
|  |  |  |  |  |  |  |  |
| Wendland et al., 2007 [59] | Universal;  Fasting blood glucose | 2-h 75-g OGTT | WHO  (Fasting ≥ 140mg/dl or 2h ≥ 160mg/dl) | Never smoked  Quit before pregnancy  Quit during pregnancy  Current smokers | 1.0  0.87 (0.65-1.16) | 1.0  0.75 (0.53-1.06)  0.53 (0.31-0.90)  0.74 (0.53-1.04) |  |

Additional file 2 - continued

| Cosson et al., 2006 [65] | Selective + universal. | 2-h 75-g OGTT | Fasting glucose > 95mg/dl, 2-hr glucose >140mg/dl, or both. | Not smoking  Smoking in pregnancy | 1.0  0.65 (0.48-0.90) |  | No adjustments. No information about exposure assessment. | |
| --- | --- | --- | --- | --- | --- | --- | --- | --- |
|  |  |  |  |  |  |  |  | |
| Berkowitz et al., 1992 [61] | Selective; 50 g GC (Screening was done according with individual obstetrician criteria) | 3-h 100-g OGTT | National Diabetes Data Group  (F ≥ 105mg/dl, 1h ≥ 190mg/dl, 2h ≥ 165mg/dl or 3h ≥ 145mg/dl). | No smoking  Smoking | 1.0  0.81 (0.53-1.24) * |  | Lack of standardization for screening criteria. Women who quit smoking before pregnancy were assigned as smokers. No adjustments. | |
|  |  |  |  |  |  |  |  | |
| Bo et al., 2001 [64] | Universal with 50g GC (≥ 140mg/dl) | 3-h 100-g OGTT | ADA  (F ≥ 95 mg/dl, 1h ≥ 180 mg/dl, 2h ≥ 155mg/dl or 3h ≥ 140mg/dl) | Former smokers  Currently smokers | 1.0  1.36 (0.79-1.72) |  | No adjustments. No information about women who quit smoking. | |
|  |  |  |  |  |  |  | |  |
| Wolf et al., 2003 [63] | Universal with 50 g GC (> 140mg/dl). | 3-h 100-g OGTT | ADA  (F > 95 mg/dl, 1h > 180 mg/dl, 2h > 155mg/dl or 3h > 140mg/dl) | Never smoking  Current smoking | 1.0  0.85 (0.68-1.36) |  | | Exclusion of higher risk women: women with GDM diagnosis early in pregnancy and with gestational hypertension or pre-eclampsia. |
|  |  |  |  |  |  |  | |  |
| Ostlund et al., 2004 [62] | Selective; based on risk factors or a random blood glucose > 126mg/dl | 2-h 75-g OGTT | WHO  (Fasting ≥ 120 or 2h ≥ 160mg/dl). | Nonsmokers  Smokers | 1.0  1.01 (0.93-1.10) |  | | No adjustments. No information about exposure assessment. |
|  |  |  |  |  |  |  | |  |
| Joffe et al., 1998 [57] | Universal; 50 g GC (≥140mg/dl) | 3-h 100-g OGTT | 1h 50g ≥ 200mg/dl or ≥ 2 abnormal values in a 3-h 100-g OGTT (F ≥ 105mg/dl, 1h ≥ 190mg/dl, 2h ≥ 165mg/dl or 3h ≥ 145mg/dl) | Never  Current smokers | 1.0  2.72 (1.61-4.59) |  | | No adjustments. No information about expossure assessment. |

GC= oral glucose challenge; OGTT= oral glucose tolerance test, F = fasting

* = calculated as Relative Risk
